# P04.64. Chinese medicine in Australia: the nature of practice and perspectives of practitioners

**DOI:** 10.1186/1472-6882-12-S1-P334

**Published:** 2012-06-12

**Authors:** A Moore, P Komesaroff, K O'Brien

**Affiliations:** 1 Monash University, Melbourne, Australia

## Purpose

With the advent of a national regulatory framework in Australia for major health care professions, the inclusion of Chinese medicine (CM) in 2012 is a significant event. Based in mandatory legislation, this is the first comprehensive national regulation of acupuncture and Chinese herbal medicine practice to occur across all states and territories in a country outside China. This report is the largest survey on primary Chinese medicine practitioners to be carried out in Australia since 1997, and the first national qualitative study to investigate the developing cultural and clinical practice dynamics within CM. This research intends not only to describe and represent the nature and perspectives of the CM professional community, in line with the CM principles of inclusion and holism, but also to contribute to mutually beneficial dialogue and collaboration with other streams of healthcare.

## Methods

Methodology includes an online and paper-distributed nationwide survey (current n=450) in English and Chinese languages, with both quantitative and qualitative items, and in-depth qualitative interviews (n=100) with practitioners and key stakeholders.

## Results

Eight key areas will be reported on: demographics; clinical practice; education; evidence in CM; regulation; professional associations; professional development; and the future of CM in Australia. Preliminary data show that the introduction of national regulation is likely to have significant impacts on the self-definition and clinical practice of individual practitioners – in particular, raising concerns regarding clinical autonomy, professional identity, western professional expectations (i.e. English language requirements and herb labelling), business and professional development, and the management of professional relationships. Significant issues surrounding education, professional entry and the definitions of CM practice are also emerging.

## Conclusion

The results present a rigorous description and systematic conceptualisation of the Australian Chinese medical workforce. The scope and size of these findings can contribute significantly towards informing the developing policy and practice of CM.

